# Recent advances in gastric cancer

**Published:** 2014

**Authors:** David Al Dulaimi

**Affiliations:** *Department of Gastroenterology, Alexandra Hospital, Redditch, UK*

*Shi M et al.* ***XB130 promotes proliferation and invasion of gastric cancer cells.*** *J Transl Med 2014;12:1*

In addition to enhancing the survival, proliferation and metastasis of thyroid cancer cells the adaptor protein XB130 may also be implicated in gastric cancer (GC). This study investigated the effects of XB130 on GC *in vitro* and *in vivo*. Silencing XB130 significantly reduced GC cell proliferation, migration and invasion in SGC7901 and MKN45 gastric adenocarcinoma cell lines. Furthermore, downregulating XB130 significantly decreased tumour growth in a xenograft nude mouse model. In both the GC cell lines and xenograft tissue XB130 silencing reduced Akt phosphorylation. In addition the expression of mesenchymal markers (fibronectin and vimentin) and metastasis-associated proteins (MMP2, MMP9, CD44) was reduced and epithelial markers (E-cadherin, α-catenin, β-catenin) was promoted following XB130 knockout. This study highlights an important role for XB130 in GC. Modulation of the epithelial-mesenchymal transition process by XB130 inhibition may represent a novel therapeutic target in GC.

*Akan Z et al.* ***Environmental radioactivity and high incidence rates of stomach and esophagus cancer in the Van Lake region: a causal relationship?*** *Asian Pac J Cancer Prev 2014;15(1):375-80*

Environmental radiation including terrestrial (radioactive nuclides in air, soil and water) and cosmic (radiation originating from space) is a relatively modifiable cancer risk factor. The high incidence of gastric and esophageal cancer in Van city, Turkey has an unclear aetiology however elevated background radiation from Van's geographical location and proximity to a nuclear power plant may be implicated. This retrospective study analysed data from 2651 cancer patients (55% male) admitted to Yuzuncu Yil Universitesi research hospital between January 2006 and December 2010 to investigate the relationship between cancer incidence and environmental radiation in Van city, Turkey. Radioactivity in air, surface soil and drinking water and indoor ^222^RN activity was evaluated.

The average annual background radiation dose in Van city (1.86 mSv/y) was less than the estimated worldwide average (2.80 mSv/y). Gastrointestinal cancers (stomach and esophagus) accounted for 27.5% of total cancer incidence in Van with stomach cancer the most common cancer type in males and females. The Ozalp and Gurpinar regions had the greatest cancer incidence (p<0.001) and highest beta radionuclide levels in drinking water. Overall beta radionuclide levels positively correlated with cancer incidence (p<0.01). This study outlines cancer incidence and background radiation in Van city, Turkey. It is unclear whether the duration that each patient resided in a particular region and therefore cumulative radiation exposure was known. In view of the multifactorial aetiology of gastrointestinal cancer it is important that other modifiable risk factors are evaluated. 

*Aviles-Jimenez F, Vazquez-Jimenez F, Medrano-Guzman R, Mantilla A, Torres J.* ***Stomach microbiota composition varies between patients with non-atrophic gastritis and patients with intestinal type of gastric cancer.*** *Sci Rep 2014;4:4202*

The risk of developing gastric cancer (GC) may be increased by any factor which encourages gastric mucosal inflammation and tissue damage. Although stomach microbiota help to modulate mucosal inflammation it is unclear whether changes in the normal microbiota population may increase the risk of GC through dysregulation of mucosal inflammation. This study evaluated stomach microbiota in five patients with non-atrophic gastritis (NAG), intestinal metaplasia (IM) and GC, respectively, who consulted the Oncology and General hospitals in the Medical Center SXXI, Instituto Mexicano del Seguro Social, Mexico City.

Bacterial genus diversity ranged from 9 to 57 in all patients and significantly differed between patients with NAG and GC (p=0.004) with a trend of reducing diversity from NAG to IM to GC. In addition the microbiota profile significantly differed between patients with NAG and GC (p<0.05) with 44 distinct operational taxonomic units (p<0.05). Changes in the gastric mucosa to a pre-cancerous and cancerous morphology may result in a less favourable environment for normal bacterial growth perhaps due to altered mucosal mucins. Whether changes in stomach microbiota are a contributory factor or an effect of local cancerous change remains unclear.

*Kim SM.* ***Clinical outcomes of endoscopic submucosal dissection for early gastric cancer in the remnant stomach after gastrectomy.*** *Ann Gastroenterol 2014;27:85-86*

Although endoscopic submucosal dissection (ESD) for remnant early gastric cancer (EGC) following gastrectomy appears an attractive therapeutic option compared with more invasive surgery there is a scarcity of outcome data. This retrospective study investigated the feasibility and effectiveness of ESD in remnant EGC in 128 patients who underwent ESD between 1997 and 2011 at the National Cancer Center Hospital, in Tokyo, Japan. The median tumour size was 13 mm (range 1-60 mm) with median procedure time 60 minutes (range 15-310 minutes). *En bloc* and curative resection was achieved in 94% and 78% of lesions respectively. The resection was non-curative in 16% of lesions. Delayed bleeding and perforation each complicated two cases (1.4%) and one patient required emergency surgery. The five-year overall survival was 87.3% and during the 4.5 y median follow-up period (range 0-13.7 y) no deaths were attributed to gastric cancer. Local recurrence was detected in two patients (1.6%) with non-curative resection margins. Metachronous cancers were found in 6.3% of patients highlighting the need for careful follow-up post procedure. Outcomes regarding cases of ulcerative EGC or lesions involving the anastamotic site or surgical suture line were not evaluated.

This study suggests ESD for remnant non-ulcerative EGC sparing the anastomotic site is an effective and safe procedure when performed by experienced endoscopists. 

*Daugherty RL et al.* ***α-Catenin is an inhibitor of transcription.*** *PNAS 2014;111(14):5260-5*

In addition to an established structural role in cell-cell adherent-type junctions α-Catenin has been shown to modify gene expression. Using several human cell types and a mouse model this study explored the effects of α-Catenin on gene transcription. α-Catenin knockdown significantly increased the rate of transcription *in vivo* and *in vitro*. The expression of several Wnt/β-Catenin responsive genes were limited by α-Catenin in three different cell lines. Nuclear accumulation of α-Catenin and inhibition of Wnt-dependent transcription required co-presence with β-Catenin. Both the β-Catenin dimerization and actin-binding domains of α-Catenin were required for inhibition of Wnt-signaling. Interestingly nuclear-targeted α-Catenin induced nuclear filamentous actin formation which correlated with reduced RNA synthesis and altered chromatin organisation. This study suggests that α-Catenin may attenuate transcription of Wnt/β-Catenin-responsive genes by modulation of nuclear actin dynamics.

*Mehta R et al.* ***Expression of energy metabolism related genes in the gastric tissue of obese individuals with non-alcoholic fatty liver disease.*** *BMC Gastroenterol 2014;14:72*

Obesity influences gene expression in non-adipose tissues including the liver although its effect on stomach tissue is poorly characterised. Given the close proximity of the stomach and liver any obesity-related changes in the gastric secretory profile may augment obesity-associated hepatic disease. This study investigated the expression of 84 energy metabolism related genes in the gastric tissue of 24 obese patients with histologically-proven non-alcoholic fatty liver disease (NAFLD).

Decreased gastric mRNA expression of CPE (-1.88, p<0.04) and IL1B (-2.5, p<0.05) was identified in patients with advanced steatosis compared to patients with mild or no steatosis. Genes encoding IL1R1 (1.99, p<0.04), OPRM1 (2.65, p<0.02), SIGMARI (3.13, p<0.03), THRB (1.94, p<0.02) and ZFP91 (3.09, p<0.01) mRNA were upregulated in the gastric tissue of patients with non-alcoholic steatohepatitis (NASH) compared to those without NASH. 21 genes identified in gastric tissue showed increased expression in advanced compared with mild hepatic inflammation (p<0.05) and expression of ADRA2B, CNR1, LEP, IL1A and OPRM1 correlated with the degree of inflammation (r>0.5, p<0.05). Gastric NTS and OPRK1 mRNA expression increased with the presence of hepatic fibrosis (p<0.02) and GHR and IL1A mRNA expression correlated with progression of fibrosis (r>0.5, p<0.05). This study characterises gastric gene expression in obese patients with concurrent NAFLD but fails to determine whether the observed changes influence development of NAFLD or whether both represent independent obesity-association effects

**Papers were prepared by:**

**Drs Luke Materacki **and** Ishfaq Ahmad, **Department of Medicine, Alexandra Hospital, Redditch, UK

